# A rare case of spontaneous inguinal faecal fistula as a complication of incarcerated Richter’s hernia with brief review of literature

**DOI:** 10.1186/s12893-015-0055-8

**Published:** 2015-05-28

**Authors:** Kuldip Singh Ahi, Ashish Moudgil, Kamna Aggarwal, Chandrashekhar Sharma, Kamlesh Singh

**Affiliations:** Department of Surgery, Rajindra Hospital / Government Medical College, 147001 Patiala, Punjab India; Department of Surgery, Govt. Medical College & Rajindra Hospital, Patiala, Punjab India

**Keywords:** Enterocutaneous fistula, Inguinal, Richter’s hernia, Spontaneous

## Abstract

**Background:**

Richter’s hernia has an early misleading presentation with tendency to strangulation due to common lack of obstructive symptoms which may lead to delay in diagnosis and hence increased mortality. Rarely inguinal Richter’s hernia may present with an uncommon complication of spontaneous fistula. The development of spontaneous faecal fistula secondary to incarcerated inguinal hernias is much rarer among the adult population as compared to the paediatric age group. Most of these fistula have been reported from developing countries like India and Nigeria and is usually the result of poverty, lack of knowledge, neglect, late presentation and lack of proper management.

**Case presentation:**

A 62 years old male presented with chief complaints of multiple openings with faecal discharge in the right groin for last 20 days with no history of constipation, trauma, and urinary or other abdominal complaints. CT scan revealed a small gut loop communicating with anterior abdominal wall. Exploratory laparotomy revealed a loop of distal ileum adherent to the internal inguinal ring which was retrieved back into the abdominal cavity. There was perforation over the loop. Resection of the segment of ileum involved was done with ileo-ileal hand sewn anastomosis and the internal inguinal ring was closed from inside of the peritoneal cavity. The openings in the skin over the inguinal region were communicated with each other and laid open due to cellulitis of the area involved and pus discharge.

**Conclusion:**

Spontaneous faecal fistula in inguinal region following rupture of strangulated Richter’s hernia especially in adults is very rare and can occur even in absence of obstructive symptoms. In presentation of any groin swelling, there is need for an early accurate diagnosis followed by prompt treatment. The delay in its diagnosis and management may result in this rare complication of spontaneous faecal fistula. This reflects the state of health care in the developing world and needs to be addressed by the concerned authorities.

## Background

Richter’s hernia is an uncommon condition in which only a circumference of the antimesenteric bowel wall is incarcerated within the hernia sac leading to ischemia, gangrene and perforation of the hollow viscus [1]. It occurs at various positions with femoral ring being the most common [2]. It has an early misleading presentation with tendency to early strangulation and the lack of obstructive symptoms which may lead to delay in diagnosis and hence increased mortality [2]. Spontaneous enterocutaneous fistula can be the presentation of an intra-abdominal pathology. Rarely inguinal hernia may present with rare complication of spontaneous faecal fistula.

Non-availability of proper medical care and unawareness of the condition are the major factors having potential for transformation of a relatively benign condition of inguinal hernia into complicated state of incarceration and strangulation. It is extremely rare to have progression of strangulation towards the development of spontaneous faecal fistula.

## Case presentation

A 62 years old male presented to surgery emergency with chief complaints of multiple openings and faecal discharge from the openings in the right groin since 20 days. One month back he noticed a small swelling in right inguinal region after exertion. A day later, he developed fever with redness over inguinal region and few days later developed multiple openings with discharge of faecal material in the right inguinal region. No history of abdominal pain, constipation or any other bowel disturbances were present. There was no history of trauma and urinary or other abdominal complaints. There was no history suggestive of an inguinal hernia in the past, and nor was there a history of any type of surgical intervention.

**Fig. 1:**
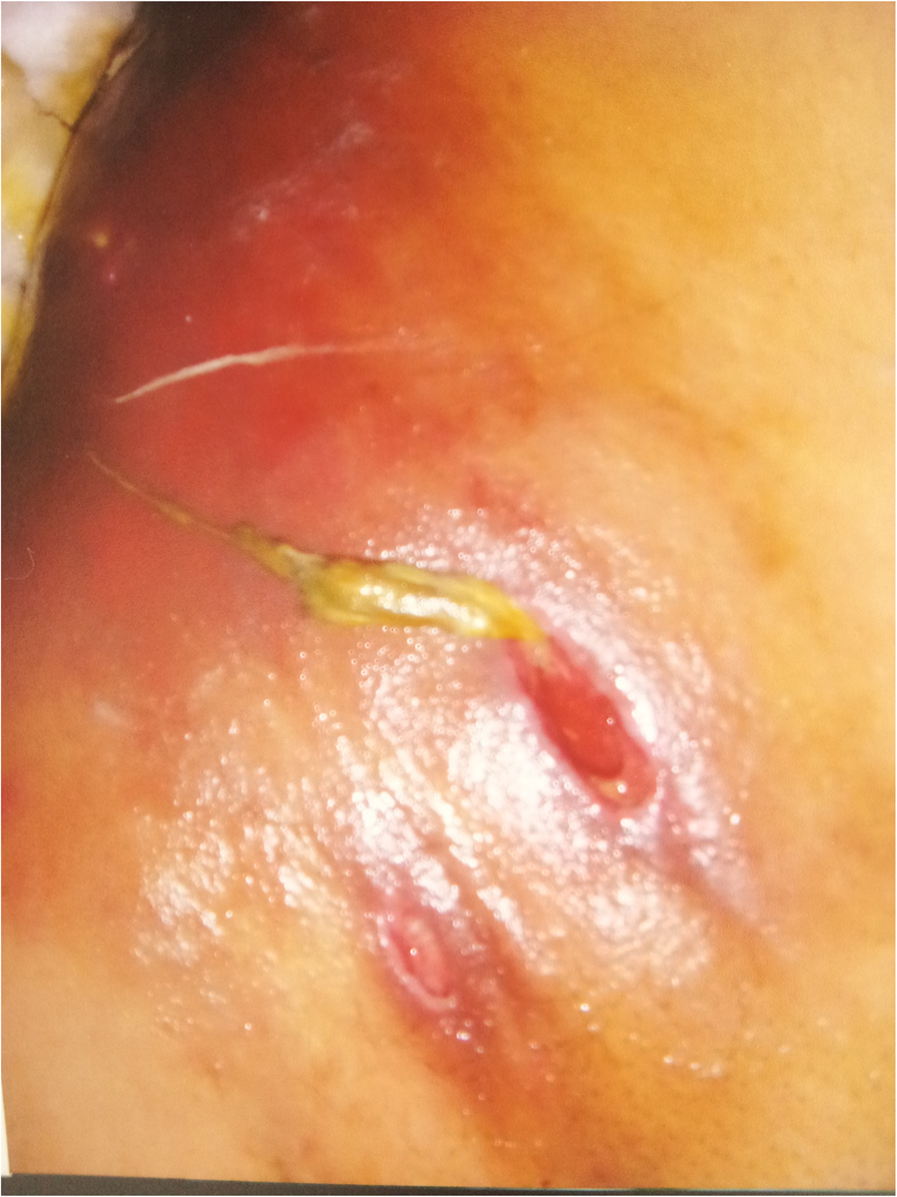
Showing multiple fistula over right inguinal region with one sinus with faecal discharge

Patient was clinically examined and multiple openings were noticed in right inguinal region with faecal and pus discharge (Fig. 1). Diagnosis of spontaneous faecal fistula was made and possibilities of appendicular abscess, tuberculosis of intestine, Crohn’s disease carcinoma colon, and actinomycosis were kept. Fistula was a low output fistula and patient was started on medical treatment and investigated thoroughly. On CT scan a small gut loop communicating with anterior abdominal wall and bilateral renal and hepatic cysts was reported with provisional diagnosis of enterocutaneous fistula and autosomal polycystic kidney disease.

**Fig. 2:**
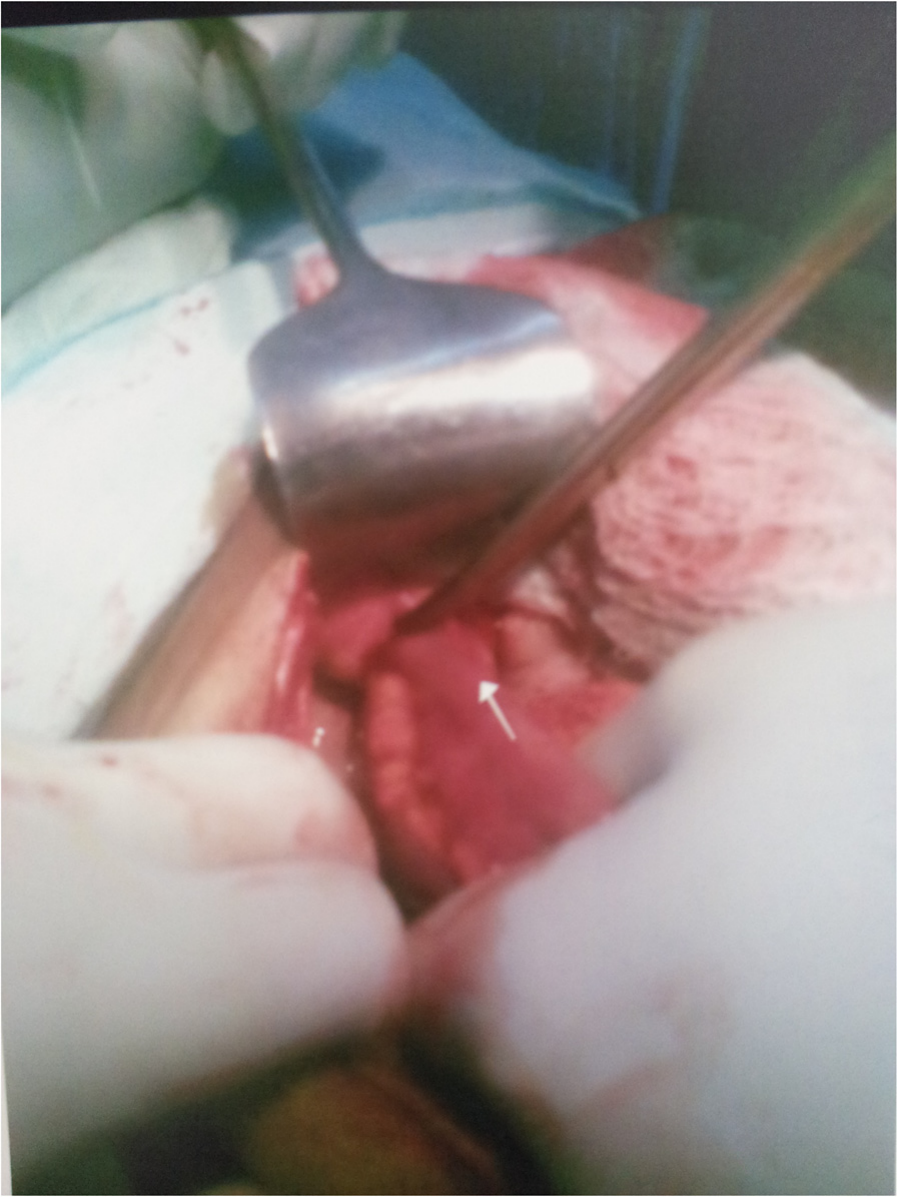
Showing loop of ileum adherent to anterior abdominal wall stuck into deep inguinal ring

**Fig. 3:**
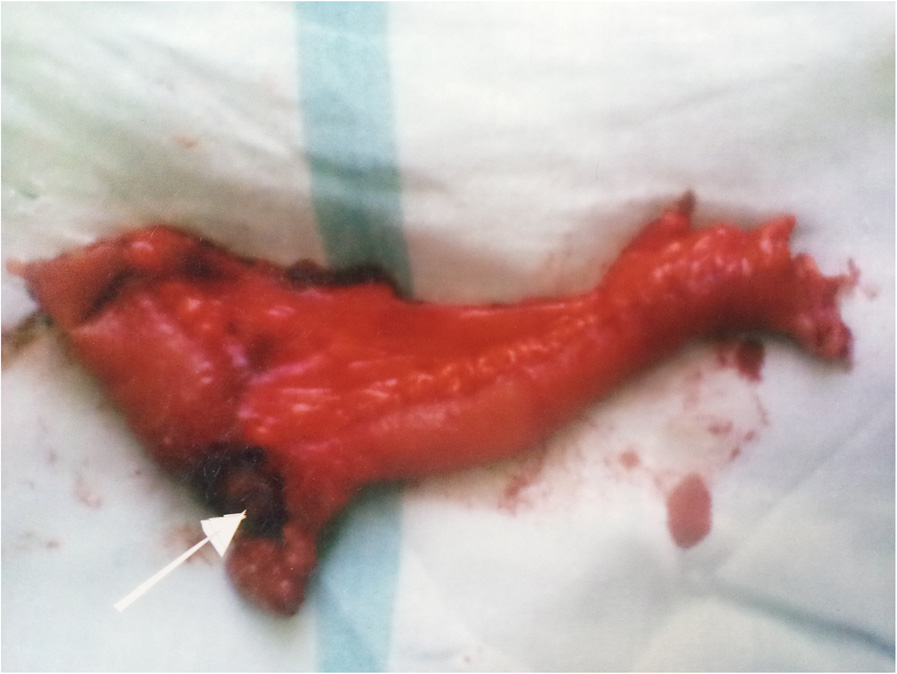
Resected segment of the involved ileum with arrow showing the perforation

There was no improvement in patient’s condition and patient had continuous faecal discharge. Exploratory laparotomy was planned. On opening the abdomen, a loop of distal ileum (about 20 cms proximal to ileocaecal junction) was found to be adherent to the internal inguinal ring which was retrieved back into the abdominal cavity **(**Fig. 2). There was perforation over the loop stuck in the deep inguinal ring. Resection of the segment of ileum (Fig. 3) involved was done with ileo-ileal hand sewn anastomosis. Mesh repair was not done for hernia defect due to faecal contamination and cellulitis. The internal inguinal ring was closed from inside of the peritoneal cavity with plicating sutures. The openings in the skin over the inguinal region were communicated with each ‘other and laid open due to cellulitis of the area involved and pus discharge. After achieving complete haemostasis and drain placement in pelvis abdomen was closed in layers. Final diagnosis of Richter’s hernia presenting as spontaneous faecal fistula was reached. Secondary suturing of the wound over inguinal region was done on 14th day. Post-operative recovery and hospital stay of patient was smooth and uneventful.

## Discussion

Richter’s hernia occurs in small hernia rings large enough to entrap the partial circumference of the bowel wall, but small enough to prevent protrusion of a loop of the intestine, with firm margins commonly occuring in the femoral ring (72 %-88 %), followed by inguinal canal (12-24 %) and the abdominal wall incisional hernias (4 %-25 %). Recently several cases have been reported at laparoscopic port insertion sites also [3]. Unusual occurrences occurs at the insertion site of the drainage tube following open abdominal surgery, as a Spigelian’s hernia, through the sacral foramen [1]. A Richter’s hernia progresses more rapidly to gangrene due to constricting ring that exerts direct pressure on the bowel wall and hence compromised blood supply. When less than two thirds of the circumference of the bowel wall is involved, the signs and symptoms of intestinal obstruction are absent. This leads to late diagnosis or even misdiagnosis, and thus it allows bowel necrosis to develop [1].

Any part of intestine may get incarcerated but most commonly involves distal ileum, caecum and sigmoid colon [2]. As only a segment of bowel is involved, luminal continuity is maintained, thus there is only partial intestinal obstruction with minimal clinical signs [2].

In 1598, Fabricius Hildanus [4], reported the earliest known case of a Richter’s hernia. Richter’s hernia is named after the German surgeon, August Gottlieb Richter, who gave the first description of this type of hernia in 1778 [1]. In 1986, Horbach found 45 Richter’s hernias among 146 strangulated hernias. Among 45 patients with Richter’s hernias, he found necrosis of the bowel wall in 31 patients; and among 101 ordinary strangulated hernias, he found bowel necrosis in only 25 patients [5].

Majority of faecal fistula occur because of surgical intervention. The development of spontaneous scrotal faecal fistula secondary to incarcerated inguinal hernias is much rarer among the adult population as compared to the pediatric age group. Most of these spontaneous faecal fistula have been reported from developing countries like India and Nigeria [6] and is usually the result of poverty, lack of knowledge, neglect, late presentation and lack of proper management [7].

Earliest in 1980 Rao et al. [8] reported 2 cases of scrotal faecal fistula in inguinal hernia, one of which was spontaneous and other was iatrogenic. In 1998, Sharma D et al. [9] reported a case of spontaneous inguinal faecal fistula as a complication of incarcerated Richter’s hernia in Maharashtra, India. In 2005 Samad A et al. [10] reported a case of congenital inguinal hernia presenting with scrotal and suprapubic faecal fistula. In 2013 a case of suprapubic faecal fistula due to Richter’s inguinal hernia was reported by Faridi S et al. [1] in Uttar Pradesh India. About 8 cases of scrotal faecal fistula have been reported in literature as a complication of inguinal hernia in paediatrics age group [6, 8, 11–17] and 8 cases of scrotal fistula as complication of inguinal hernia in adults [7, 10, 18–24] have been reported in literature. Onakpoya et al. [25] from Nigeria reported the case of a neglected Richter’s inguinal hernia presenting with perforation and Fournier’s gangrene. Three cases of spontaneous perforation of Richter’s inguinal hernia with Fournier’s gangrene were reported by Guzzo et al. [26] in 2007 from the United States of America.

A case of port site Richter’s hernia presenting intestinal obstruction following laparoscopic surgery for the inguinal hernia was reported by Wegener et al. [27] Faecal fistula may also result from the placement of a prosthetic material in the peritoneal cavity [20]. Leslie et al. [28] from the United Kingdom reported the case of a spontaneous faecal fistula secondary to a Littre’s hernia. Repeated treatment of scrotal hernias by native doctors has also been reported as a cause of multiple urinary and faecal fistula in one study from Nigeria [29]. The incision of inguinal hernia by herbalists as well as intervention by quacks has been reported as the cause of faecal fistula in adults by Nwabunike [21].

In 1956 Gillespie classified patients with Richter’s hernia into three clinical groups according to the presentation of this disease. The obstructive group characterised by nausea, vomiting, peritonitis and constipation which if untreated leads to shock. The second group was post necrotic group characterised by strangulation with necrosis and perforation causing enterocutaneous fistula. The third group was dangerous group which includes patients with minimal abdominal signs [30]. This group has the maximum morbidity and mortality owing to delay in diagnosis.

Friedman stated that death is the inevitable fate of the patient with a strangulated hernia, unless the resulting obstruction is relieved by operative interference, spontaneous reduction of the hernia or the formation of an external faecal fistula. He further states: “The pathologic changes taking place in the formation of a faecal fistula are in the beginning like those of any strangulated hernia: first, there is an exudation of a bloody fluid into the hernial sac, and with impairment of integrity of the bowel, infection of the fluid. As the sac wall becomes infected and edematous, the bowel perforates into, and then through the sac, thereby involving the external hernia coverings. Infection and necrosis then spread rapidly through the subcutaneous tissues, and finally rupture occurs externally through the skin, forming an external faecal fistula” [31].

The presence of fistula allows decompression of the bowel and temporary relief of the intestinal obstruction. Unrelieved strangulation will, however, increase the likelihood of septic complications and mortality associated with intestinal obstruction. Strangulation may be associated with testicular ischemia and infarction [16, 23]. This would necessitate orchidectomy. Therefore, urgent surgical exploration with bowel resection and end-to-end anastomosis is necessary to avert this [22]. Surgically, a ‘high’ approach gives more generous access when strangulation of hernia has occurred/suspected or when an intra-abdominal pathology was suspected as in this case.

## Conclusion

This case report highlights one of the very few cases of spontaneous faecal fistula in inguinal region following rupture of strangulated Richter’s hernia especially in adults and stress upon suspicion of Richter’s hernia even in absence of obstructive symptoms presenting as faecal fistula and in presentation of any groin swelling, the need for an early accurate diagnosis followed by prompt treatment. The delay in its diagnosis and management may result in various complications including this rare complication of spontaneous faecal fistula. This reflects the state of health care in the developing world and needs to be addressed by the concerned authorities [7].

## Consent

Written informed consent was obtained from the patient for publication of this Case report and any accompanying images. A copy of the written consent is available for review by the Editor of this journal.
